# A Mouse Model of Otitis Media Identifies HB-EGF as a Mediator of Inflammation-Induced Mucosal Proliferation

**DOI:** 10.1371/journal.pone.0102739

**Published:** 2014-07-17

**Authors:** Keigo Suzukawa, Julia Tomlin, Kwang Pak, Eduardo Chavez, Arwa Kurabi, Andrew Baird, Stephen I. Wasserman, Allen F. Ryan

**Affiliations:** 1 Division of Otolaryngology, University of California, San Diego School of Medicine and VA Medical Center, La Jolla, California, United States of America; 2 Division of Trauma, Department of Surgery, University of California, San Diego School of Medicine and VA Medical Center, La Jolla, California, United States of America; 3 Division of Allergy-Immunology, Department of Medicine, University of California, San Diego School of Medicine and VA Medical Center, La Jolla, California, United States of America; The Hospital for Sick Children and The University of Toronto, Canada

## Abstract

**Objective:**

Otitis media is one of the most common pediatric infections. While it is usually treated without difficulty, up to 20% of children may progress to long-term complications that include hearing loss, impaired speech and language development, academic underachievement, and irreversible disease. Hyperplasia of middle ear mucosa contributes to the sequelae of acute otitis media and is of important clinical significance. Understanding the role of growth factors in the mediation of mucosal hyperplasia could lead to the development of new therapeutic interventions for this disease and its sequelae.

**Methods:**

From a whole genome gene array analysis of mRNA expression during acute otitis media, we identified growth factors with expression kinetics temporally related to hyperplasia. We then tested these factors for their ability to stimulate mucosal epithelial growth *in vitro*, and determined protein levels and histological distribution *in vivo* for active factors.

**Results:**

From the gene array, we identified seven candidate growth factors with upregulation of mRNA expression kinetics related to mucosal hyperplasia. Of the seven, only HB-EGF (heparin-binding-epidermal growth factor) induced significant mucosal epithelial hyperplasia *in vitro*. Subsequent quantification of HB-EGF protein expression *in vivo* via Western blot analysis confirmed that the protein is highly expressed from 6 hours to 24 hours after bacterial inoculation, while immunohistochemistry revealed production by middle ear epithelial cells and infiltrating lymphocytes.

**Conclusion:**

Our data suggest an active role for HB-EGF in the hyperplasia of the middle ear mucosal epithelium during otitis media. These results imply that therapies targeting HB-EGF could ameliorate mucosal growth during otitis media, and thereby reduce detrimental sequelae of this childhood disease.

## Introduction

Otitis media (OM) is one of the most common childhood infections and the leading cause of visits to physicians in the pediatric population [1]. Although most cases of OM are treated without difficulty, up to 20% of children progress to persistent or recurrent acute OM or chronic OM. These conditions have presented major therapeutic challenges in the management of the disease [2]. Furthermore, they may lead to many long-term complications, including conductive and sensorineural hearing loss [3], impaired speech and language development [4], impaired academic achievement [5], and irreversible middle ear (ME) disease [6]. In the developing world, OM is a much more serious problem. Worldwide, OM is responsible for an estimated 28,000 annual deaths and contributes more than half of the world’s burden of serious hearing loss (∼175 million cases) [7]. A better understanding of the pathophysiology of the ME inflammatory response during OM may direct us toward potential new therapies for this disease.

Hyperplasia of the ME mucosa is an important component of OM, involving substantial cell proliferation and differentiation [8]. Hyperplasia contributes to the deleterious sequelae of OM, including the production of mucous secretions of ME effusions [9]. It is also involved in fibrosis and other permanent damage that can occur in repeated and/or chronic OM [10]. The regulation of mucosal hyperplasia in the ME is, therefore, of substantial clinical significance.

The mechanisms that control ME mucosal hyperplasia are poorly understood. However, in most complex organisms, the growth and differentiation of dividing cells are controlled in part by peptide growth factors [11]–[14]. These endogenous, biologically active substances are constructed locally and released in a paracrine fashion at their site of action [13].

Previous investigations have supported the involvement of growth factors in the proliferation and differentiation of the ME mucosa during OM. For example, fibroblast growth factors 1 and 2 (FGF 1 and FGF 2) appear to play a role in the growth, hyperproliferation, and differentiation of the stromal compartment of the ME mucosa during OM, but not the mucosal epithelium [15], [16]. While some studies of epidermal growth factor (EGF), betacellulin, and keratinocyte growth factor (KGF) have implicated them in mucosal epithelial proliferation [17], [18], these represent only a fraction of the growth factors that can influence epithelial cells that might be expressed in the mucosa during OM. We hypothesized that a comprehensive survey of growth factor expression during ME infection, and kinetic analysis to link expression to mucosal hyperplasia, would allow the identification of candidates that underly the growth of the mucosal epithelium, which could then be tested for growth promotion. The hypothesis was tested in widely used mouse and rat models of OM. These species are recognized as sharing the major characteristics of this disease with humans [19]–[21].

The goal of the present study was to identify growth factors that are modulated during bacterial OM and which demonstrate the abililty to regulate mucosal epithelial growth. To do so, we used a whole-genome gene array technique across an entire episode of acute OM, focusing on growth factor genes that were expressed just prior to mucosal hyperplasia. We identified seven factors with appropriate expression kinetics. These factors were then tested for their ability to enhance the growth of the ME mucosal epithelium *in vitro*. Also, we confirmed *in vivo* expression and distribution of HB-EGF, which induced proliferation in the mucosal epithelium *in vitro*.

## Materials and Methods

### Animals

Microarray studies were performed on C57BL/6:CB F1 hybrid mice. To obtain sufficient tissue, additional *in vitro* and *in vivo* studies were performed on Sprague-Dawley rats. All animal studies were performed in strict accordance to the recommendations in the Guide for the Care and Use of Laboratory Animals of the National Institutes of Health (NIH) and carried out in strict accordance with an approved Institutional Animal Care and Use Committee (IACUC) protocol from the San Diego VA Medical Center. All surgeries were performed under anesthesia, and all efforts were made to minimize suffering. Animals were housed in SPF conditions using standard, IACUC-approved husbandry procedures and veterinary care.

### DNA microarray

Expression of selected genes involved in angiogenesis was evaluated in mice by DNA microarray. This method was chosen over qPCR or protein assays since we were able to assess the responses of essentially all mouse genes simultaneously. Age-matched (60–90 days) C57BL/6:CB F1 hybrid mice were purchased from Jackson Laboratories (Bar Harbor, ME, USA). F1 hybrid mice were used to reduce the potential effects of recessive mutations that are common in inbred strains. Forty mice per time point were inoculated bilaterally with the *Haemophilus influenzae* strain 3655 (nontypeable, biotype II; NTHi), in 5 µL at a concentration of 10^5^–10^6^/mL as described previously [22], [23]. Uninoculated animals (time 0) served as controls. All of the mucosal tissue that could be recovered from the ME was harvested from 20 mice at each of the following intervals: 0 hours (0 h, no treatment), 3 h, 6 h, 24 h, 2 days (2d), 3d, 5d and 7d after NTHi inoculation, and pooled. The pooled tissue was homogenized in TRIzol (Life Technologies, Carlsbad, CA USA) and total RNA was extracted. Total RNA quality was assessed using the RNA 6000 Labchip Kit on the Agilent 2100 Bioanalyzer for the integrity of 18S and 28S ribosomal RNA. The mRNA was reverse transcribed using a T7-oligodT primer, then transcribed *in vitro* using T7 RNA polymerase to generate biotinylated cRNA probes that were hybridized to 2 Affymetrix MU430 2.0 microarrays. This procedure was duplicated for each time point to obtain a second, independent replicate. Thus each postinoculation time point represents 2 separate samples consisting of 20 mice each, and 4 Affymetrix arrays, for a total of 320 mice and 16 arrays in the study. Raw intensity data was median normalized and statistical differences in gene transcript expression levels were evaluated using a variance-modeled posterior inference approach (VAMPIRE) [24]. Specific genes were assessed at individual time points, after Bonferonni correction for multiple tests, using Genespring GX 7.3 (Agilent Genespring Technologies, Santa Clara, CA USA). Of the 136 genes included in the Silicon Genetics GO category: growth factor activity (Genespring), all genes that were upregulated 5-fold or more at or before 24 h after NTHi inoculated were examined for evidence in the literature of documented growth promotion in epithelial cells.

### Middle ear mucosal tissue explants

Male Sprague-Dawley rats weighing between 200 and 250 g were anesthetized; ME bullae were inoculated bilaterallary with approximately 20 µL of NTHi solution at a concentration of 10^5^–10^6^/mL as described previously [25], [26]. A sham operation control group received saline in the ME. 48 h after inoculation, the animals were deeply anesthetized and sacrificed, and the ME bullae were surgically extracted bilaterally from the rats. The bullae were immediately placed in a Petri dish with warm phosphate-buffered saline (PBS). The ME mucosae were surgically removed and divided into roughly square tissue explants ranging from 0.5 to 1.0 mm^2^ using a surgical blade in culture media consisting of a mixture of Dulbecco’s modified Eagles medium and Ham’s F12 medium (3∶1) supplemented with fetal calf serum (3.5%), hydrocortisone (0.4 mg/mL), isoproterenol (10^−6 ^mol/L), penicillin (100 U/mL), and streptomycin (100 mg/mL). It is known that explants of various sizes from 0.5 to 2.0 mm^2^ showed no effect on growth of the cellular skirt from the explant. The explants were individually transferred, epithelium uppermost, into single wells of a 24-well Falcon cell culture plate containing 170 mL of the previously described culture media. They were placed in an incubator at 37°C with 5% CO_2_ for 24 h and allowed to adhere to the culture plate surface. After 24 h, 180 mL of culture media was added to each well and replaced in the incubator for 48 h. On day 4, each well was individually examined with a microscope under magnification. All wells with healthy, attached explants were randomly divided into 4 groups of 12 explants each, determined by power analysis to provide adequate statistical power to detect a 20% increase in growth. Twenty-one groups of 12 explants were then cultured in media supplemented with one of seven growth factors in three different concentrations; the soluble form of HB-EGF (heparin-binding-epidermal growth factor; 0.5, 5, and 50 ng/mL; Sigma Ardrich, St. Louis, MO USA), amphiregulin (1, 10, 100 ng/mL; R&D Systems, Minneapolis, MN USA), epiregulin (0.5, 5, 50 ng/mL; R&D Systems); inhibin βa (0.5, 5, 50 ng/mL; Abcam, Cambridge, MA USA); Gdf15, (1, 10, 100 ng/mL; R&D Systems); LIF (0.5, 5, 50 ng/mL; AbD Serotec, Raleigh, NC USA); or Cxcl1 (0.5, 5, 50 ng/mL; R&D Systems), and three groups of 12 explants each received media alone to serve as negative controls. The plates were replaced in the incubator and on days 6 and 8, the culture media with the appropriate growth factors was changed.

Each explant was photographed daily until day 10 with the aid of a dissecting microscope (Leica, Germany) at the same magnification and standard exposure conditions. The area of each explant was measured daily using ImageJ software (National Institute of Health, Bethesda, MD USA). For statistical analysis, the normal distribution of the data was confirmed, and ANOVA was performed with a criterion P value of 0.05, and Bonferroni correction for repeated analyses.

### Western blot

To assess the relative expression of HB-EGF protein during OM infection, 18 male Sprague-Dawley rats (200–250 g) were injected with NTHi in the same manner described above, using the same batch of bacteria and on the same day. The animals were sacrificed and both ME bullae surgically extracted at 3 h, 6 h, 12 h, 24 h, 3d, 5d or 7d after inoculation. Because more tissue could be harvested from the ME during the peak of OM, three rats were sacrificed for each of the 3 h, 6 h, 5d and 7d time point samples, while two rats were sacrificed at 12 h, 24 h and 3d sample. Each bulla was immediately placed in a 60 mm Petri dish containing PBS, and ME mucosa was surgically removed intact. Mucosae from four to six MEs were pooled, depending upon the time point. Six ME mucosae from three uninoculated rats (0 h) were also dissected to serve as negative controls. The samples were immediately placed in 150 to 400 µL (depending upon the amount of tissue collected) of T-Per Tissue Protein Extraction Reagent (Thermo Scientific, Rockford, IL USA) in 1X phosphatase/protease inhibitors (Roche, Indianapolis, IN USA) for homogenization and lysis. Samples were sonicated for 10 min. The homogenates were then centrifuged at 10,000 G for 10 min at 4°C and the pellets discarded. Protein concentrations were equalized by spectophotometry using a NanoDrop 2000 (Thermo Scientific). Equal quantities of protein were then resolved on Bis-Tris Mini Gels 4–12% (Life Technologies) and electrotransferred to polyvinylidene diflouride membranes (Bio-Rad, Hercules, CA USA). The membranes were then cut just below the 38 kDa marker, and the top halves probed with an antibody against the internal control protein actin (BD Transduction Laboratories, San Diego, CA USA). The lower portion of each membrane was blocked with 1X casein in TBS-Tween [50 mM Tris-HCl (pH 7.4), 150 mM NaCl, 0.05% Tween 20] for 90 min at room temperature. The blots were incubated with goat polyclonal anti-HB-EGF antibody (R&D Systems) at a concentration of 1∶500 in blocking buffer containing 0.5X casein and 5.5% BSA overnight at 4°C. The same blots were then incubated with horseradish peroxidase conjugated secondary antibodies at a concentration of 1∶20,000, and the protein visualized with chemiluminescent detection (GE Healthcare, Piscataway, NJ USA). After chemiluminescent exposure, membranes were placed inside a dark chamber, an autoradiography film was laid over the membranes to capture light emission. They were exposed and developed. The intensity of the bands corresponding to HB-EGF and actin were quantified using ImageJ software. Band intensity for the HB-EGF protein was corrected at each time point for the intensity of the corresponding actin band and then expressed as a relative intensity compared with the control tissue. Western blotting was replicated three times with independent biological samples. Relative intensity data were analyzed for significance using Kruskal-Wallis nonparametric ANOVA and Mann-Whitney U-test.

### Immunohistochemistry

To assess the histological distribution of HB-EGF in the normal and infected ME, male Sprague-Dawley rats (200–250 g) were sacrificed 3 h, 6 h, 12 h, 24 h, 2d, 3d, 5d or 7d after inoculation with NTHi as described above. Untreated animals (0 h) served as controls. After deep anesthesia with rodent cocktail (a mixture of ketamine and xylazine hydrochloride at 100 mg/mL and acepromazine at 10 mg/mL) delivered intramuscularly, rats were transcardially perfused with saline followed by 4% paraformaldehyde solution. The ME bullae were immediately removed, post-fixed for 24 h, and decalcified in solution containing 4% paraformaldehyde and 8% EDTA for 2 weeks. Specimens were then embedded in OCT compound and serial frozen sections (6 µm) were cut on a cryostat. Sections were dried overnight. Slides were washed with PBS, and endogenous peroxidase activity was quenched with 0.3% H_2_O_2_ for 30 min. Then, following 30 min of blocking with 2.5% normal horse serum, sections were incubated with goat polyclonal anti-HB-EGF antibody (Santa Cruz Biotechnology, Santa Cruz, CA USA) at a concentration of 1∶50 overnight. For detection of the primary antibody, ImmPRESS Polymer Detection Kit and DAB substrate (Vector Laboratories, Burlingame, CA USA) were used. Finally, sections were counterstained with hematoxylin and mounted. Three animals were evaluated at each time point.

## Results

### Gene array analysis identifies seven growth factor candidates

**Figure 1:**
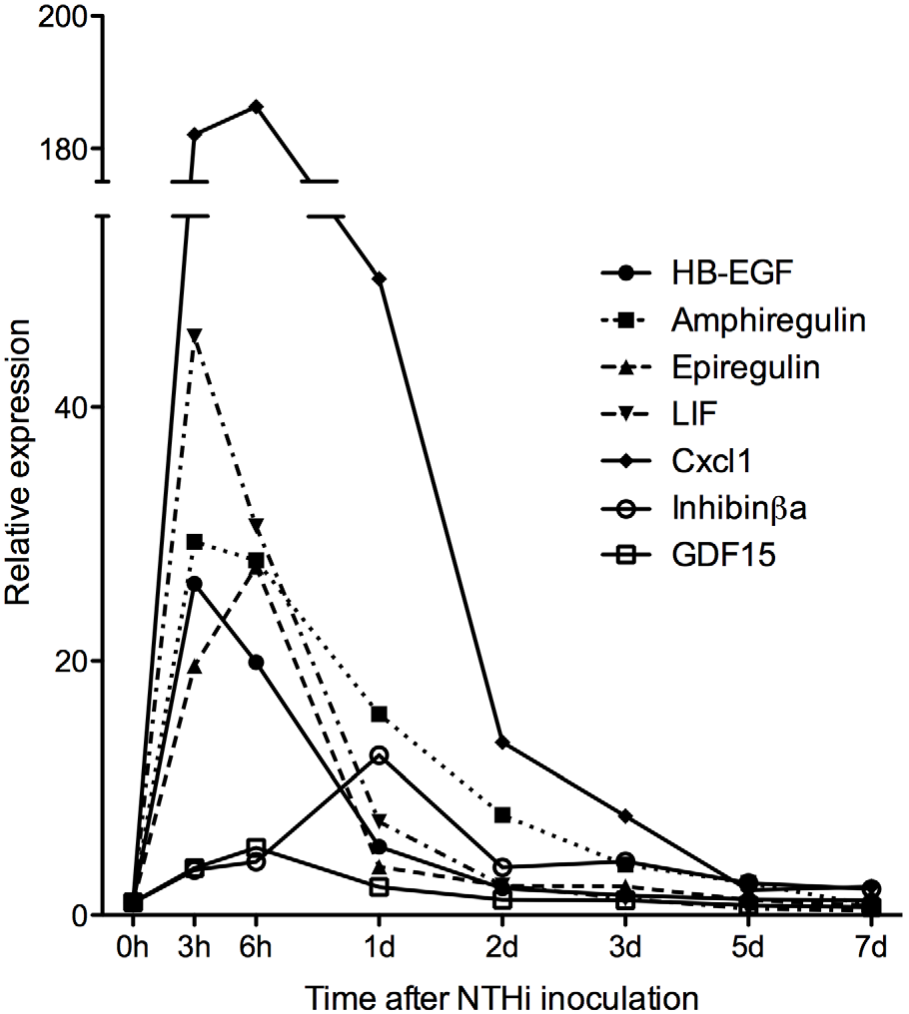
Expression of mRNA encoding growth factors identified for their potential to influence ME mucosal hyperplasia, based on the kinetics of upregulation in excess of 5-fold, assessed by gene array in the mouse ME during NTHi-induced acute OM. Data are expressed as relative increase over untreated ME mucosa (0 h). Details of variability and statistical signficance are provided in Table S1.

From a whole-genome array analysis of mRNA expression during acute OM ***in vivo***, we identified seven growth factors that are known to be active on epithelial cells, and that were strongly (>10X) upregulated within 24 h of NTHi inoculation, as illustrated in Figure 1. These factors were judged most likely to be involved in ME epithelial hyperplasia, which begins at about 24 h [27], [28]. HB-EGF, Epiregulin, LIF, Cxcl1, and GCF15 all demonstrated significant upregulation of expression that was greatest between 3 h and 6 h postinoculation. Amphiregulin showed the highest increase in fold expression, as well as the most prolonged upregulation, peaking at 6 h postinfection and remaining high through 24 h. While inhibin βa had a minimal response at 3–6 h after inoculation, it was sharply upregulated at 24 h after infection. In all cases, fold expression levels had returned to pre-inoculation values by 5d, coinciding with the return of the ME epithelium to near-normal thickness.

### Middle ear mucosal tissue explants

**Figure 2:**
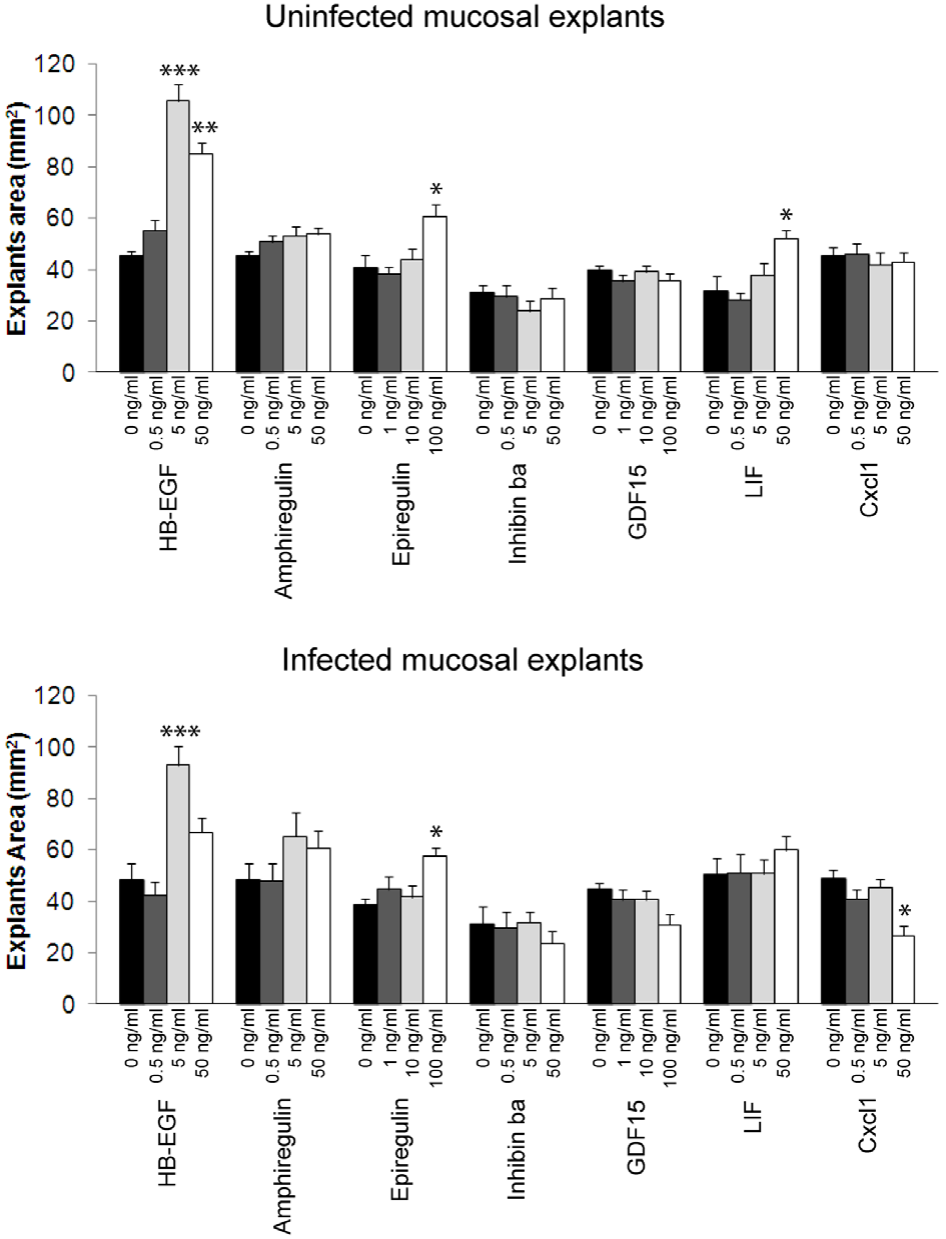
*In vitro* growth of ME mucosal explants treated with seven different growth factors identified by gene array analysis for their potential role in hyperplasia during acute OM. Each factor was tested on control (uninfected) mucosa as well as tissue from rats inoculated with NTHi. The graph represents the means of area ± standard errors (SE) of each experimental group on the 10^th^ and final day of culture. (n = 12 at each group). For this and subsequent figures, * = p<.05, ** = p<.01, *** = p<.001.

**Figure 3:**
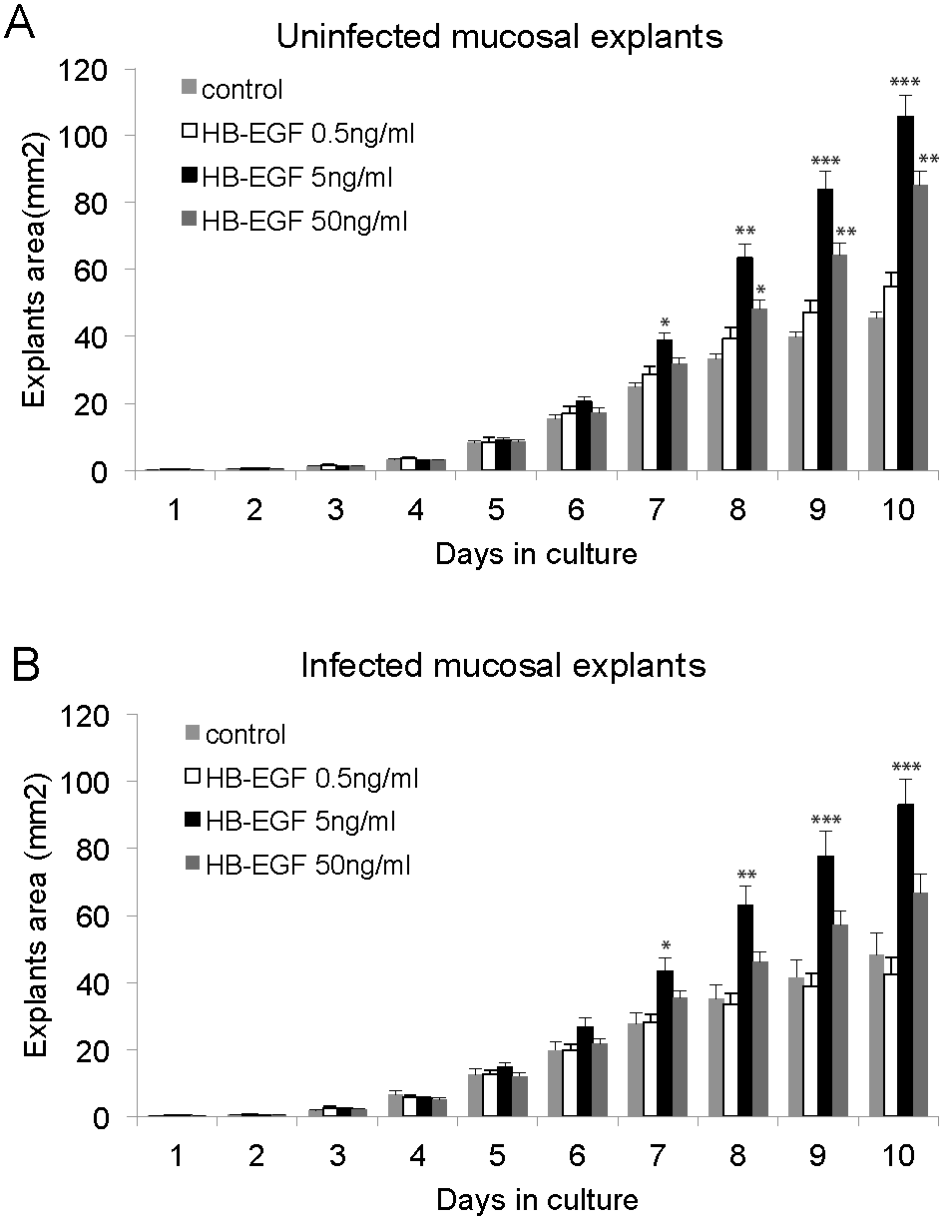
Daily change in surface area of mucosal explants with administration of rHB-EGF. (**A**) Uninfected mucosal explants were cultured in the presence of various concentrations of rHB-EGF (0.5, 5, or 50 ng/ml). A negative control without rHB-EGF was identically treated. (**B**) Infected mucosal explants which were harvested 48 h after bacterial inoculation were cultured in the presence of various concentrations of rHB-EGF (0.5, 5, or 50 ng/mL), or in unsupplemented media (control). Values are means ± standard errors (SE) (n = 12 at each group).

Each of the seven growth factors was tested at 3 concentrations related to its published ED50 on other tissues, and 12 explants were evaluated at each concentration. All explants maintained a healthy appearance and remained firmly attached to the well surface throughout the 10 days of the study. Figure 2 illustrates the effect of growth factors on the explant surface area. The control explants (0 ng/mL) provided a baseline for growth and differentiation of epithelial cells in each experiment. Three of the factors tested, including Epiregulin, Inhibin βa, and GCF 15, had no significant stimulatory or inhibitory effect on mucosal growth *in vitro,* for either the infected or uninfected explants, despite the fact that their mRNA was upregulated during AOM. Amphiregulin demonstrated a mild though statistically significant (P<.05) effect on both uninfected and infected mucosa, but only at the highest concentration of 100 ng/mL. LIF induced a postive growth effect at the highest concentration on uninfected explants (P<.05), but did not show a significant effect on infected mucosa. Cxcl1 proved to be slightly inhibitory of growth on infected explants at 50 ng/mL (P<.05), though this effect was not observed in non-infected mucosa. In contrast to this modest or absent stimulation, HB-EGF in both infected and non-infected explants demonstrated a greater than two-fold increase in mucosal area over untreated tissue at the 5 ng/mL concentration (Figs. 2 and 3, P<.001). This response was somewhat attenuated at the highest concentration of 50 ng/mL, though still highly significant (P<.01) for the uninfected mucosa.

### HB-EGF protein is strongly expressed in the infected ME mucosa

**Figure 4:**
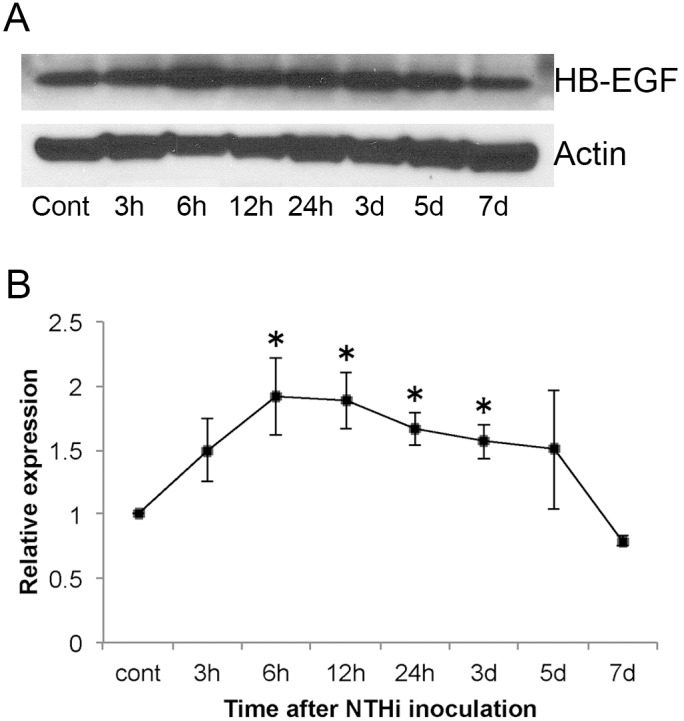
Western blot of HB-EGF in the ME mucosa. (**A**) Blot of HB-EGF expression in the ME at various postinoculation times. Total protein was equalized in all lanes and compared using an actin protein standard. (**B**) Quantitative analysis of HB-EGF shows a peak in relative expression at 6 h with continued upregulation through 3d, followed by a gradual decrease to control (uninoculated) levels.

Figures 4A and 4B illustrate the levels of HB-EGF protein observed in Western blots of the ME mucosa at various *in vivo* postinoculation time points. Using ImageJ software, the intensity of the bands developed in Western blotting (Fig. 4A) were quantified. With actin as an internal control, HB-EGF levels were normalized and expressed as a relative frequency as compared with control levels (Fig. 4B). HB-EGF protein expression demonstrated the strongest increase at 6 h postinfection with NTHi, and showed continued upregulation of relative intensity through 3d. By 7d, the level of HB-EGF protein had returned to control levels, corresponding with the clearing of infection appreciable during dissection. Of note, the large variance observed at 5d postinoculation is likely due to the incorporation of mucosal tissue from rats that had not yet successfully begun to clear the infection, which is typically observed at this time point.

### HB-EGF is expressed by both mucosal cells and leukocytes

**Figure 5:**
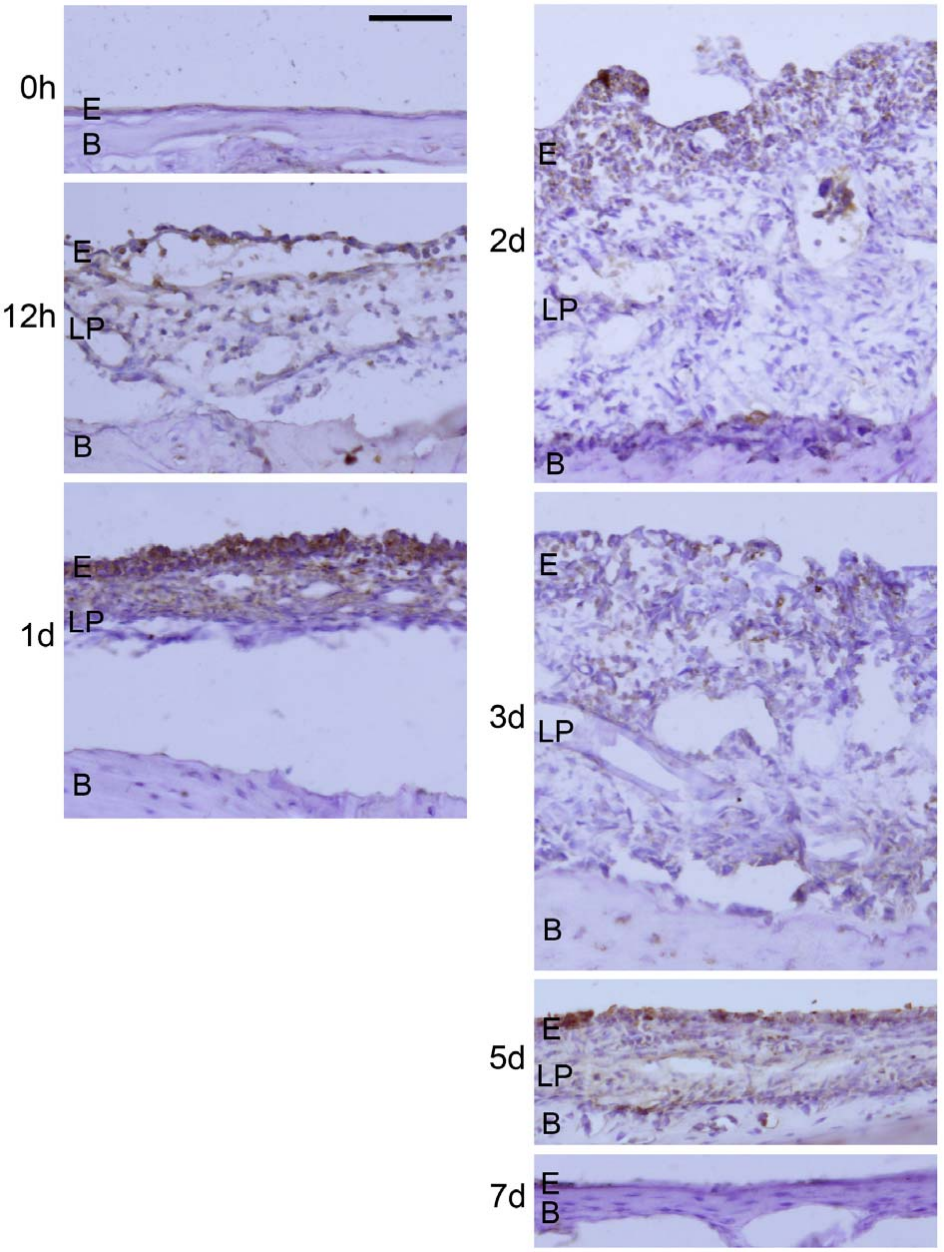
Immunohistochemistry of HB-EGF in the ME mucosa during OM. Frozen sections of the rat ME were immunostained with a primary antibody directed against HB-EGF. Sections of uninfected ME mucosa (0 h) and infected ME mucosa (12 h, 24 h, 2d, 3d, 5d or 7d after infection) were processed. Positive reaction product (brown) was observed from 12 h to 5d postinoculation. Labeling was observed primarily in the epithelial layer of ME mucosa and in scattered leukocytes within the tissue or within the ME lumen. E: epithelial layer, LP: lamina propria, B: bone of ME bulla. Scale bar represents 100 µm.

Figure 5 illustrates immunohistochemical staining of HB-EGF in the bacterially infected ME mucosa of rats. The mucosa was thin in control MEs (0 h), then progressed to hyperplasia after infection. Mucosal thickness peaked at 2d and 3d, then normalized by 7d. Immunostaining for HB-EGF was not obvious in the control (0 h) mucosa. By 12 h, positive staining was seen, which persisted to 5d. Staining was not observed on 7d postinfection. Positive staining was primarily observed in the epithelial layer and in inflammatory cells in ME exudates.

## Discussion

In this study, we evaluated growth factors for their potential to induce mucosal epithelial hyperplasia during the course of bacterial OM. The major findings of this study are as follows: 1) using gene arrays, seven growth factor candidates with upregulation of mRNA temporally related to hyperplasia in OM were identified; 2) of these seven, HB-EGF demonstrated the strongest stimulatory effect on mucosal growth *in vitro*; 3) HB-EGF protein was highly expressed in the ME from 6 h to 24 h after bacterial inoculation and was produced by ME epithelial cells as well as infiltrated leukocytes *in vivo*.

Whole-genome DNA microarray analysis revealed seven factors with mRNA expression kinetics related to the hyperplastic response of the mucosa in OM. HB-EGF, amphiregulin, and epiregulin are growth factors that belong to the EGF family, and are known to have mitogenic effects on epithelial cells in other systems [29]–[31]. Activin and GDF-15 are members of the TGF-beta family. Activin consists of a dimer of the inhibin βa subunit and also has mitogenic effect on epithelial cells [32]. GDF-15 is known to be highly expressed in various kinds of malignant tumors; its effect on cell proliferation remains somewhat controversial [33]. LIF is a polyfunctional molecule that has been reported to stimulate the proliferation of an array of cell types [34]. Cxcl1 is a cytokine that was previously named growth-related oncogene alpha (GROα), and has been reported to have a mitogenic effect in malignant melanoma cells [35].

Using an *in vitro* model, we applied these growth factors to normal mucosal explants as well as those infected with NTHi. Only amphiregulin had been tested previously with the same *in vitro* model on uninfected ME mucosa [18], and our repeat evaluation yielded data consistent with these previous results. Despite significant mRNA upregulation determined via DNA microarray analysis for all seven factors, we only observed strong and consistent mitogenic potential for HB-EGF. There are several possibilities to account for these results. First, while all of these growth factors are upregulated during the infection process, they may not participate in mucosal growth. As our explant model is limited to the evaluation of epithelial cells, these factors could potentially influence other mucosal component(s), such as the stroma or vascular tissue. They may also play a role in the proliferation of leukocytes that infiltrate the ME from circulation. Furthermore, it must be acknowledged that our *in vitro* experimental model is an imperfect representation of the *in vivo* environment of the ME during OM. Of course, it is possible that the process of extracting tissue from the middle ear and placing it in culture has altered the mucosa, making it unresponsive to growth factors that influence its responses *in vivo.*

HB-EGF was originally detected in conditioned medium from the U937 macrophage-like cell line and later found to be a member of the EGF family of growth factors [36], [37], which is consistent with our observation of HB-EGF protein in infiltrating leukocytes. HB-EGF is synthesized as a transmembrane protein (proHB-EGF) and can be cleaved at the plasma membrane by metaloproteinases to yield soluble HB-EGF (sHB-EGF). sHB-EGF exerts its mitogenic effects by binding to and activating EGF receptor subtypes ErbB1 (the canonical EGF receptor) and ErbB4 [38]. Mitogenic activity of sHB-EGF was initially demonstrated using smooth-muscle cells and fibroblasts [39]–[41], but other cell types, such as keratinocytes, hepatocytes, kidney tubule cells, and gastrointestinal epithelial cell lines, also respond to this factor [42]. HB-EGF is expressed in a variety of tissues and in a large number of cultured cells including vascular endothelial and smooth muscle cells, inflammatory cells, skeletal muscle fibers, renal mesangial cells, keratinocytes, and tumor cells. Although normal tissues express relatively low levels of HB-EGF mRNA, expression increases in response to various types of tissue damage, including hypoxia, wounding, and partial resection [43]–[47]. Particularly relevant to the present study, HB-EGF can also be upregulated in response to bacterial infection or stimulation by LPS [48]–[53].

Despite expansive knowledge regarding the role of HB-EGF in other tissues and cell lines, the effect of this factor on ME mucosa has, to our knowledge, never been considered. On mucosal explants *in vitro*, HB-EGF demonstrated a strong stimulatory effect comparable to that of EGF, which was tested previously in our laboratory [18]. However, in our array datasets the expression of the gene encoding EGF itself was strongly downregulated during OM (data not shown), arguing against a role for this factor. HB-EGF, at a concentration of 5 ng/mL, had the largest effect on mucosal growth in both infected and uninfected conditions. This is compatible with the reported effective dose of HB-EGF on previously tested cell lines, which varies from 1–50 ng/mL [54], [55]. The stimulatory effect of HB-EGF was somewhat reduced at 50 ng/mL, though it remained significant in explants from uninfected MEs. During the growth of an explant, cells must not only proliferate but also migrate away from the center to achieve observed growth. At a concentration of 50 ng/mL of HB-EGF, we speculate that an imbalance between proliferation and migration may account for the explants’ decreased size as compared with 5 ng/mL. Alternatively, 50 ng/mL may downregulate the receptors for HB-EGF, as has been shown for many growth factors including EGF receptors [56].

As noted above, a transmembrane form of proHB-EGF is cleaved to release sHB-EGF, which interacts with ErbB1 and ErbB4 to stimulate cell proliferation [38]. In our gene array datasets, ErbB1 mRNA is significantly upregulated (2–3X) at 24 h after NTHi inoculation, while ErbB4 mRNA is not regulated, emphasizing a role for ErbB1 in HB-EGF ME signaling. However, it should be noted that HB-EGF has additional functions mediated by the interactions with other proteins that could potentially contribute to its role in OM. Soluble HB-EGF interacts with N-arginine dibasic convertase (NRDc) to mediate cell migration [57]. NRDc mRNA displayed modest but significant (∼2X) upregulation at 24 h in our array data (data not shown). Cleavage of transmembrane proHB-EGF also releases the cytoplasmic domain, which can interact with promyelocytic leukemia zinc finger (PLZF) protein [58] to promote cell cycle progression. However, we found PLZF mRNA to be significantly downregulated during OM, suggesting that this mechanism of action is unlikely to contribute to ME mucosal hyperplasia. Finally, uncleaved pro-HB-EGF can interact with the chaperone regulator BCL2-associated athanogene (Bag1), to promote cell survival [59]. However, Bag1 mRNA was not significantly altered in our array data.

Western blot data showed that HB-EGF protein was present in the normal ME mucosa, but was significantly upregulated in the infected ME from 6 h to 24 h, with a peak at 6 h after inoculation. Taking into account time for translation of mRNA, this increase is consistent with DNA microarray data that demonstrated a peak in expression of HB-EGF mRNA at 3 h after bacterial inoculation. Although the array analysis was conducted in a mouse OM model, it should be translatable to the rat ME, as they have nearly the same chronological inflammatory response following bacterial inoculation [60]. Cleavage of transmembrane HB-EGF by metaloproteinases is required for activation, and the large influx of neutrophils that occurs within the first 24 h of bacterial inoculation [27], [28] would provide a rich source of MMP9 [61]. Considering that ME mucosal hyperplasia peaks around 2–3d after inoculation, the result of the Western blot implies that HB-EGF, which has a strong mitogenic effect and is highly expressed in early stages of infection, could play an important role in inducing mucosal hyperplasia [62]. Finally, immunohistochemistry demonstrated that HB-EGF is produced by epithelial cells of the ME mucosa as well as by infiltrated cells. Similar results have been reported in inflammatory oral mucosa [63], and neutrophils have been shown to produce HB-EGF in response to GM-CSF [64]. It has also been found that IL-1β, a major inflammatory cytokine for which we observed mRNA to be strongly upregulated early in OM, promotes release of sHB-EGF [65]. Although no detailed mechanism regarding upregulation of HB-EGF in the ME following bacterial infection has been clarified, innate immune responses seem likely to play an important role in the induction of this growth factor in OM.

## Conclusions

The results of this study indicate significant involvement of HB-EGF in the hyperplastic response of the ME mucosa during bacterial OM. HB-EGF was one of seven growth factors active on epithelial cells for which expression kinetics were consistent with a role in mucosal hyperplasia. It was also the only factor which stimulated the growth of normal or previously infected mucosa *in vitro*. Expression of HB-EGF protein was confirmed *in vivo*. These data are consistent with a dominant role for HB-EGF in regulating the growth of the ME mucosal epithelium during OM. As HB-EGF has also been linked to epithelial-mesenchymal transformation [66], it may also function in the de-differentiation of epithelial cells that seems likely to occur prior to mucosal hyperplasia. Given the close similarity between OM in animals and humans [8], [9], [67], [68], our results have potentially important implications in the treatment of this disease. Our data also have implications for other respiratory epithelial sites at which mucosal growth contributes to pathology, including the lung and nasopharynx [69], [70]. Administration of anti-HB-EGF compounds or blockage of EGF receptors may mitigate mucosal growth and survival of hyperplastic tissue in the ME, thereby reducing irreversible complications of OM. In the future, studies with HB-EGF-null animals may further elucidate HB-EGF function in OM.

## Supporting Information

Table S1 **Growth Factor Genes.**
(DOC)
