# Supplementary material for: A Mouse Model of Otitis Media Identifies HB-EGF as a Mediator of Inflammation-Induced Mucosal Proliferation
Source: PLoS One. 2014 Jul 17;9(7):e102739. doi: 10.1371/journal.pone.0102739 (PMC4102546; doi:10.1371/journal.pone.0102739)
Supplement: Table S1 — Growth Factor Genes. (DOC) [file pone.0102739.s001.doc]

# SUPPLEMENTARY TABLE 1. Growth Factor Genes

| **Time:** | **0h** | **3h** | **6h** | **24h** | **2d** | **3d** | **5d** | **7d** |  |
| --- | --- | --- | --- | --- | --- | --- | --- | --- | --- |
|  |  |  |  |  |  |  |  |  |  |
| ***Areg*** 1421134_ | |  |  |  |  |  |  |  |  |
| **Fold Exp** | 1.0 | 29.4 | 27.9 | 15.8 | 7.9 | 4.0 | 2.5 | 0.8 |  |
| **Range** | 1.0 – 1.0 | 24.0 – 35.9 | 16.6 – 46.9 | 15.6 – 16.0 | 7.2 – 8.7 | 3.9 – 4.1 | 1.8 – 3.6 | 0.3 – 1.9 |  |
| **P-Value** | 0.99 | 0.04 | 0.1 | **0.003** | **0.03** | **0.008** | 0.23 | 0.84 |  |
|  |  |  |  |  |  |  |  |  |  |
| ***Cxcl1*** 1419209_ | |  |  |  |  |  |  |  |  |
| **Fold Exp** | 1.0 | 182.1 | 186.3 | 50.1 | 13.6 | 7.8 | 2.0 | 2.2 |  |
| **Range** | 1.0 – 1.0 | 176.8 – 187.6 | 160.2 – 216.6 | 48.1 – 52.2 | 11.1 – 16.7 | 6.6 – 9.2 | 1.5 – 2.7 | 2.0 – 2.5 |  |
| **P-Value** | 0.99 | **0.004** | **0.02** | **0.007** | 0.05 | 0.05 | 0.26 | 0.09 |  |
|  |  |  |  |  |  |  |  |  |  |
| ***Ereg*** 1419431_ | |  |  |  |  |  |  |  |  |
| **Fold Exp** | 1.0 | 19.6 | 27.4 | 3.8 | 2.3 | 2.3 | 1.2 | 0.8 |  |
| **Range** | 0.9 – 1.1 | 17.1 – 22.5 | 19.7 – 38.3 | 3.2 – 4.5 | 1.8 – 3.0 | 1.9 – 2.8 | 1.1 – 1.3 | 0.5 – 1.1 |  |
| **P-Value** | 0.96 | **0.03** | 0.06 | 0.07 | 0.2 | 0.15 | 0.37 | 0.56 |  |
|  |  |  |  |  |  |  |  |  |  |
| ***Gdf15*** 1418949_ | |  |  |  |  |  |  |  |  |
| **Fold Exp** | 1.0 | 3.7 | 5.3 | 2.2 | 1.2 | 1.2 | 0.8 | 0.6 |  |
| **Range** | 0.9 – 1.1 | 3.4 – 4.1 | 4.8 – 5.9 | 1.9 – 2.5 | 1.0 – 1.4 | 0.6 – 2.3 | 0.6 – 1.1 | 0.6 – 0.7 |  |
| **P-Value** | 0.98 | **0.04** | **0.04** | 0.11 | 0.45 | 0.87 | 0.57 | 0.08 |  |
|  |  |  |  |  |  |  |  |  |  |
| ***Hbegf*** 1418350_ | |  |  |  |  |  |  |  |  |
| **Fold Exp** | 1.0 | 26.1 | 19.9 | 5.4 | 2.1 | 1.6 | 1.2 | 1.2 |  |
| **Range** | 0.8 – 1.2 | 23.2 – 29.3 | 17.0 – 23.3 | 5.3 – 5.5 | 1.8 – 2.5 | 1.3 – 1.9 | 1.2 – 1.3 | 1.0 – 1.4 |  |
| **P-Value** | 0.95 | **0.02** | **0.03** | **0.009** | 0.15 | 0.24 | 0.18 | 0.6 |  |
|  |  |  |  |  |  |  |  |  |  |
| ***Inhba*** 1422053_ | |  |  |  |  |  |  |  |  |
| **Fold Exp** | 1.0 | 3.6 | 4.2 | 12.6 | 3.8 | 4.2 | 2.5 | 2.1 |  |
| **Range** | 0.9 – 1.2 | 2.9 – 4.4 | 3.6 – 5.0 | 10.8 – 14.6 | 3.7 – 3.8 | 3.7 – 4.8 | 2.0 – 3.3 | 1.8 – 2.3 |  |
| **P-Value** | 0.95 | 0.12 | 0.07 | **0.04** | **0.004** | 0.05 | 0.17 | 0.1 |  |
|  |  |  |  |  |  |  |  |  |  |
| ***Lif*** 1421207_ | |  |  |  |  |  |  |  |  |
| **Fold Exp** | 1.0 | 45.5 | 30.6 | 7.4 | 2.3 | 1.3 | 0.5 | 0.3 |  |
| **Range** | 0.9 – 1.1 | 43.2 – 48.1 | 27.3 – 34.3 | 7.0 – 7.8 | 1.9 – 2.9 | 1.0 – 1.7 | 0.2 – 1.1 | 0.3 – 0.4 |  |
| **P-Value** | 0.96 | **0.009** | **0.02** | **0.02** | 0.15 | 0.48 | 0.54 | 0.06 |  |
|  |  |  |  |  |  |  |  |  |  |
